# Prevalence and Burden of Nausea and Vomiting in Pregnant Women: Final Analysis of the PURITY Survey

**DOI:** 10.3390/jcm15041365

**Published:** 2026-02-09

**Authors:** Valeria Maria Savasi, Serena Tinti, Francesca Praticò, Veronica Bonaldo, Marika Ylenia Rovetto, Roberta Panniello, Dionisio Franco Barattini, Elena Casolati, Elena Piccolo, Mario Mangrella, Marco Liberati, Mariavittoria Locci, Irene Cetin

**Affiliations:** 1 Department of Woman, Mother and Neonate, Vittore Buzzi Children’s Hospital, University of Milan, 20154 Milan, Italy; valeria.savasi@asst-fbf-sacco.it (V.M.S.); serena.tinti@unimi.it (S.T.); pratico.francesca05@gmail.com (F.P.); veronica.bonaldo@unimi.it (V.B.); 2 Department of Neuroscience and Reproductive and Dentistry Sciences, University of Naple Federico II, 80131 Napoli, Italy; marika.rovetto@gmail.com (M.Y.R.); mariavittoria.locci@unina.it (M.L.); 3 Obstetrics-Gynecology Clinic, SS. Annunziata Hospital, University of Chieti, G. D’Annunzio, 66100 Chieti, Italy; r.panniello@gmail.com (R.P.); marco.liberati@unich.it (M.L.); 4 Opera CRO, a TIGERMED Group Company, 300209 Timișoara, Romania; barattini@operacro.com; 5 Private Practice of Obstetrics and Gynecology, 20122 Milan, Italy; elecaso55@gmail.com; 6 Italfarmaco SpA, Medical Affairs Department, 20126 Milan, Italy; e.piccolo@italfarmacogroup.com (E.P.); m.mangrella@italfarmacogroup.com (M.M.); 7 Obstetric Unit “Clinica Mangiagalli”, Foundation IRCCS Ca’ Granda Ospedale Maggiore Policlinico, 20122 Milan, Italy; 8 Department of Clinical Sciences and Community Health, University of Milan, 20122 Milan, Italy

**Keywords:** nausea and vomiting in pregnancy, prevalence, pregnancy, maternal distress

## Abstract

**Background/Objectives**: Nausea and vomiting of pregnancy (NVP) are common and potentially debilitating symptoms of early pregnancy. However, data on their prevalence and impact in Italy are limited. This survey aimed to assess the frequency of NVP among Italian pregnant women and to evaluate its impact on quality of life. **Methods**: The survey was conducted in three public university hospitals in Italy during two separate periods. Women with multiple pregnancies or who conceived by medically assisted reproduction were excluded. The Questionnaire for Pregnancy Period, including the Pregnancy-Unique Quantification of Emesis (PUQE), was administered during a face-to-face interview at 18–22 weeks of gestation, coinciding with the morphological ultrasound. A structured telephone follow-up interview was performed within 14 days after delivery. This report presents the final analysis of all valid, completed questionnaires. **Results**: A total of 532 pregnant women were included (mean age 32.7 ± 4.9 years); 277 (52.1%) were primiparous. Overall, NVP was reported by 66.4% of participants. Nausea alone occurred in 28.0% of women, while nausea overall (with or without vomiting) was reported by 64.3%. Vomiting alone was reported by 2.1% and vomiting overall by 38.4% of participants. Symptoms began at a mean gestational age of 7.0 ± 2.8 weeks, lasted 9.7 ± 5.1 weeks, and were still present at the first interview in 30.3% of cases. More than half of the affected women (51.0%) reported limitations in daily activities, particularly work-related activities. **Conclusions**: This final analysis confirms a high prevalence of NVP among Italian pregnant women and highlights its substantial negative impact on quality of life. Systematic screening and appropriate management strategies should be considered in routine prenatal care.

## 1. Introduction

Nausea with or without vomiting during the early stages of pregnancy (NVP) affects approximately 50–80% of pregnant women [1,2]. In Italy, indirect evidence of NVP prevalence has been provided by a web-based survey conducted among nearly one thousand women as part of a broader multinational cross-sectional study carried out in 2011–2012 [3,4].

Epidemiological studies from other countries, including Norway and the United States, have reported comparable prevalence rates and have consistently highlighted the substantial impact of NVP on women’s daily lives [5,6]. Several investigations have further explored the relationship between NVP and quality of life (QOL) [7,8]. A recent systematic review confirmed that NVP is among the most frequently reported factors associated with impaired physical, mental, and social dimensions of QOL in pregnant women [9]. Moreover, multiple studies have shown that QOL—particularly physical functioning, work performance, and overall maternal well-being—progressively declines with increasing NVP severity [10,11,12,13,14,15,16], regardless of geographical setting.

Despite the high and consistent prevalence of NVP worldwide, management strategies vary considerably, potentially reflecting cultural differences and attitudes toward medication use during pregnancy. Given the limited data available from Italy, we initiated a dedicated survey in 2021 to assess the prevalence of NVP and its impact on maternal QOL (study acronym Purity). Our interim analysis conducted after the enrollment of 200 women reported an NVP prevalence of 65.5% and demonstrated a significant association between symptom severity and the impairment of daily activities and social relationships [17].

Here, we present the results of the final analysis of the Purity survey, focusing on the prevalence of NVP, its impact on quality of life, and patterns of medication use as the primary study outcomes.

## 2. Materials and Methods

### 2.1. Study Design

This is a multicenter, open, prospective, non-comparative cross-sectional study that makes use of a survey and adheres to the ICH E6 (R2) GCP Guidelines, the International Ethical Guidelines for Biomedical Research Involving Human Subjects, and the Declaration of Helsinki (2013). The study protocol final version 3.0, dated 1 January 2021, was registered at www.clinicaltrials.gov public registry (no. NCT04926727) and approved by the local Italian Ethics Committees (EC) Milano Area 1; Chieti (Pescara) Università “D’Annunzio”; and Napoli Università “Federico II” on 24 February 2021, 17 June 2021, and on 16 April 2021, respectively. The study was performed at the following centers: Ospedale dei Bambini “Vittore Buzzi” Milano (Northern Italy); Policlinico Universitario “Federico II” Napoli (South Italy); Presidio Ospedaliero “SS.ma Annunziata” Chieti (Central Italy). All recruited subjects signed an informed consent.

### 2.2. Study Population

The inclusion criteria were as follows: (i) women of Caucasian origin; (ii) physiological pregnancy between 18 and 22 weeks; (iii) ability to understand the questionnaires and communicate adequately with the interviewer; (iv) written informed consent. The exclusion criteria included the following: (i) twin pregnancies; (ii) medically assisted reproduction. Patients were prospectively recruited in the hospital at the time of morphological ultrasound.

### 2.3. Survey Objectives and Outcomes

The first interview (Questionnaire for Pregnancy Period) was conducted between 18 and 22 weeks of pregnancy and consisted of 39 questions, including the Pregnancy-Unique Quantification of Emesis (PUQE) questionnaire [18] (Table 1).

The same cohort of women was interviewed a second time using a post-pregnancy questionnaire comprising nine questions. The interviews were conducted immediately after birth or no later than 14 days after delivery. The two questionnaires were described in the interim analysis article [17] and are provided as Supplementary Materials.

The final analysis was performed on all data collected from both questionnaires. Data were collected from 553 valid questionnaires from subjects enrolled in the three involved sites. The survey began on 15 December 2021, when the first subject completed the first questionnaire, and ended on 19 August 2024, when the last subject completed the second questionnaire. The database was locked on 21 March 2025.

### 2.4. Monitoring and Data Management

The interviews were conducted by the investigators using a PC tablet and the paper-and-pen interview (PAPI) technique to conduct the interviews. The data collected were subsequently entered into the Electronic Data Collection (EDC) system, which was accessible only to authorized individuals through a secure web connection. The Microsoft Forms program “https://forms.microsoft.com (accessed on 28 November 2025)” was utilized for this purpose. The EDC forms complied with GDPR and HIPAA regulations. The SSAE 18 SOC 1 and 2 reports attested to the data storage services’ compliance with EU data residency regulations. Opera CRO, an independent contract research organization based in Timisoara (Romania), handled the monitoring activities, data management, and statistical analysis.

### 2.5. Sample Size and Statistical Analyses

The calculation of the sample size and statistical methods were reported in the interim analysis [17]. Statistical analyses were performed using R statistical software v 4.2.0. The impact of the previous interim analyses on the study results increased the margin of error of 7% (due to the limited sample size of 232 subjects). In this final analysis the margin of error was reduced to 4.2%.

## 3. Results

### 3.1. Subject Enrolment and Analyses

**Figure 1:**
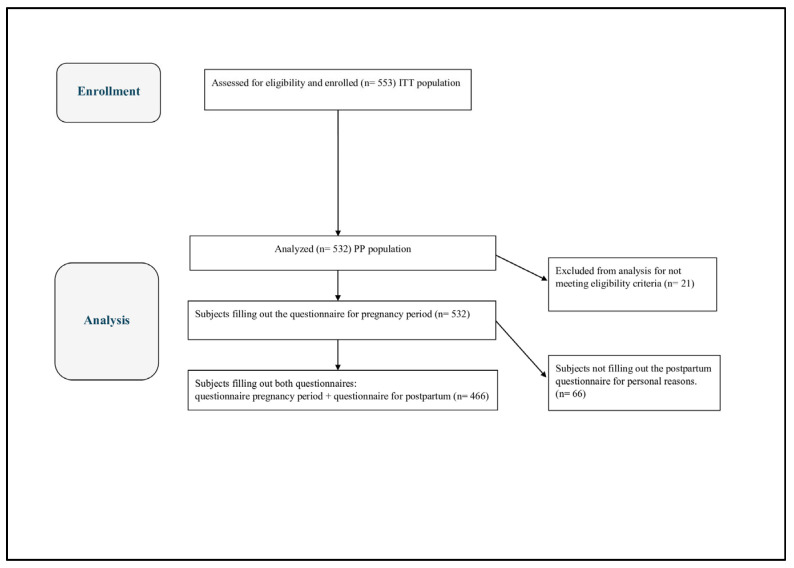
Subject disposition—Consort diagram.

A total of 553 participants completed the initial questionnaire and were considered as Intention to Treat (ITT) population; 532 subjects were considered evaluable (PP population in Figure 1), including 199 from center 01 (Milano), 190 from center 02 (Napoli), and 143 from center 03 (Chieti-Pescara).

As many as 277 (52.1%) of all the subjects recruited were primiparous (with a 57.5% parity), and the mean age was 32.7 years (median 33 years, range 30–36 years). Table 2 displays the demographic and baseline characteristics by Italian geographical regions.

The majority of women were employed (90.6%, 396 women out of 437, the total number of women who declared their working status). Statistically significant differences were observed in the city of residence, education levels, and work status among geographical regions (*p* < 0.001). No significant difference was observed in the age group distribution of women across geographical regions (*p* = 0.037). Table 3 illustrates the maternal-related data for all subjects by Italian geographical region. Notably, statistically significant differences were observed for being primiparous and the type and sex of care provider, with *p*-values of 0.029 and <0.001, respectively.

### 3.2. NVP Prevalence Rate and Characteristics

The prevalence of NVP was calculated as the ratio of subjects who experienced these symptoms during the 18–22-week window and the total number of surveyed women. The resulting NVP prevalence was 66.4% (353 out of 532 subjects), as shown in Table 4.

The prevalence of nausea alone was 28.0% (149 out of 532), while nausea overall (with or without vomiting) was 64.3% (342 out of 532). The prevalence of vomiting alone was 2.1% (11 out of 532) and vomiting overall (with or without nausea) was 38.4% (204 out of 532). Based on a margin of error of 4.2% for the current survey, 95% confidence of NVP prevalence lies between 62.2% and 70.6%, with a midpoint of 66.4% (±4.2%). The prevalence rates of NVP were consistent across various demographic factors, such as age, geographical region, population of the city of residence, education, and work status (*p* > 0.05 in all cases). Out of 353 women reporting NVP, 218 had moderate symptoms and 123 experienced mild symptoms. Only 12 women had severe symptoms. The relationship between NVP symptoms and their severity (Table 5) revealed that women who experienced both nausea and vomiting were more likely to have severe symptoms (only the 12 women experiencing both symptoms reported severe symptoms).

Symptoms typically began between weeks 4 and 20 (mean of 7.0 weeks), with the range for severe symptoms being shorter (between weeks 4 and 10). The severity of symptoms, scored by PUQE as mild, moderate, or severe, dramatically influenced their duration in months or weeks (*p* < 0.001) and their duration beyond the first trimester of pregnancy (*p* < 0.001). Finally, 41.7% of women with severe NVP complained that their symptoms were still ongoing at the time of the first interview. This percentage was lower among subjects with moderate (34.9%) and mild (21.1%) NVP.

### 3.3. Quality of Life

Several items of the questionnaire evaluated the potential consequences of NVP symptoms, specifically their effects on daily life and overall QOL (QOL by NVP severity). Several women (15 cases, 4.2% of the total) required emergency care due to their NVP symptoms. The Fisher–Freeman–Halton test revealed a statistically significant association between symptom groups and emergency visits (*p* = 0.008). In particular, subjects experiencing both nausea and vomiting were more likely to have accessed the emergency department (7.3%) compared to those with nausea only (0.7%) or vomiting only (0.0%). NVP had a statistically significant impact on pregnant women’s relationship with their partners (*p* = 0.001) and on social relationships (*p* = 0.001); the impact was statistically different across the NVP type (nausea only, vomiting only, or both). Table 6 presents the impact on the daily activities of pregnant women in relation to NVP severity (*p* < 0.001).

In addition (Table 7), NVP had a statistically significant impact differently across the NVP severity on pregnant women’s relationships with their partners, social relationships, and work activities (*p* = 0.001 in all cases).

Must be underlined that the severity of NVP is strongly associated with the women’s decision to take time off from work because of their symptoms (*p* < 0.001) and also on the decision to have another child (*p* = 0.020). The impact of NVP symptoms consequences (i.e., emergency room visits, hospitalizations, daily activities, social relationships, work activities, and relationships with their partners) was not significantly different between geographical regions (*p* > 0.05 in all cases).

### 3.4. Medications

Of the women included in the study, 131 out of 532 (24.6%) are reported to have taken a total of 146 medications (with 16 different types) for symptoms related to NVP. Furthermore, it is noteworthy that 12 women were prescribed multiple medications. The most frequently administered medication was the combination of doxylamine succinate and pyridoxine hydrochloride [19], which was recently introduced in the Italian market. This combination was utilized in 66.4% of cases (97 out of 146). The second most prevalent medication was a granulate composed of sodium citrate, potassium citrate, thiamine, riboflavin, vitamin B6, and citric acid, which was used in 18.5% of cases (27 out of 146). The medications intake exhibited no significant variation across Italian geographical regions (*p* = 0.383). The fixed association of doxylamine succinate and pyridoxine hydrochloride was identified as the most frequently utilized medication for managing NVP, irrespective of its severity, ranging from mild to severe manifestations.

### 3.5. Gestation and Postpartum Questionnaire Data

A total of 466 women completed the postpartum questionnaire: 179 (38.4%) from Northern Italy, 146 (31.3%) from Central Italy, and 141 (30.3%) from Southern Italy. Analysis of birth-related variables (newborn sex, birth complications, and gestational age) showed no association with NVP severity as measured by the PUQE score (*p* > 0.05 for all). Conversely, several factors were significantly associated with preterm birth, including birth complications, small-for-gestational-age infants, and lower birth weight (*p* < 0.001 for all). Pregnancy-related outcomes were reported in 28.8% of women with NVP (90/313) and in 32.0% of women without NVP (49/153) with no statistically significant difference between the two groups (χ^2^ test, *p* = 0.54). In the NVP population a statistically significant difference in the distribution of perinatal outcomes across PUQE severity categories was observed (χ^2^ = 21.5, *p* < 0.001). Complications were more frequently reported among women with moderate and severe PUQE scores compared with those with mild scores (Table 8).

Even though the analysis showed a correlation between women from southern Italy and preterm delivery (*p* < 0.002) and shorter gestational age (269.5 ± 15.6 days; *p* < 0.001), these events were likely caused by multiple factors and not solely by NVP.

Earlier delivery and shorter gestational duration were also more frequent in women reporting vomiting alone compared with those experiencing nausea alone or nausea plus vomiting (*p* = 0.019 and *p* = 0.026, respectively).

Maternal characteristics (age, residence, education, employment, parity, and care provider) showed no significant association with gestational term (*p* > 0.05), except for lower education, which was linked to higher preterm birth rates (*p* = 0.007).

Neither NVP duration, onset, nor its clinical outcomes (emergency visits, hospitalizations, daily activity limitations) significantly affected gestational term. In contrast, NVP symptoms had a notable impact on women’s work activity and sick leave, with significant differences observed across preterm, term, and post-term births (*p* = 0.009 and *p* = 0.014, respectively).

All evidence reported in the analysis of the gestation and postpartum questionnaire data was present when considering the entire population that completed the questionnaire (466 subjects) and when focusing on the subset of 313 women with NVP who filled out the questionnaire.

### 3.6. Safety

No adverse events were reported.

## 4. Discussion

### 4.1. Overview of Study Findings

This paper presents the results of a survey concerning NVP in a large Italian population. We report a high prevalence of NVP, ranging from 61.6% to 70.2%, with a moderate severity of symptoms affecting 66.4% of women, and a symptom duration of approximately 10 weeks. Previously published data have shown that approximately 70% of women worldwide report having NVP [4,5,20], although actual rates vary widely. The data of the present analysis indicate that the prevalence of NVP in Italy ranges from 62.2% to 70.6%, in agreement with the results obtained in different regions [4,5,6]. This result is similar to that obtained in our previously published interim analysis, in which 65.5% of the women surveyed had NVP and its prevalence in Italy was estimated to be between 58.5% and 72.5%, with a margin of error of 7% [17]. In this survey, the prevalence of nausea alone was 28.0%, and the prevalence of total nausea (with or without vomiting) was 64.3%. The prevalence of vomiting alone was 2.1%, and total vomiting (with or without nausea) was 38.4%. In addition, our study showed that women’s quality of life was negatively impacted by NVP symptoms, which significantly affected daily activities and social relationships, confirming what previous studies [8,9,11,13] and also our interim analysis had already shown. Similarly, the analysis demonstrated the impact of NVP severity on relationships with partners and work activities. Moreover, women who experienced both nausea and vomiting were more likely to visit the emergency department (7.3%).

International data indicate wide variability in NVP management due to cultural and prescribing differences [4]. In China, treatment rates for severe NVP were low [21], while in the United States, about 10% of affected women required pharmacotherapy [22], and one-third of those with excessive vomiting had multiple medical encounters [23]. A French survey found that 93% of general practitioners prescribed antiemetics but considered them effective in only 30% of patients, while dietary modification was frequently recommended (68%) but rarely successful [24]. A recent European web-based study reported pharmacological treatment in only 16% of women [25]. Although drug use remains limited, most studies highlight the need for better clinical support and counseling, as pharmacist consultation alone did not improve NVP symptoms or quality of life [24]. In Italy, data on NVP prevalence, impact, and treatment are scarce. The only previously available information derived from a survey among 157 gynecologists (study Purity Light) [26], showing that 54.8% of the interviewed medical doctors indicated a preference for prescribing medications also in mild cases of NVP to avoid the potential progression to hyperemesis, while 26.7% limited the prescription only to severe cases.

Of the 532 women analyzed in the present survey, 131 (24.6%) used NVP medications, primarily the combination of pyridoxine hydrochloride and doxylamine succinate (66.4% of cases). In another study, Di Iorio et al. [26] reported that 54.8% of gynecologists elected to prescribe drugs also in mild cases of NVP, and 26.7% administered drugs only to severe cases. These discrepancies can be attributed to a different selection of the study subjects. In the current study, the population analyzed consisted of any patient who visited the three clinical centers (Milano, Napoli, and Chieti-Pescara), with no advertising methods employed to encourage women to participate in the survey. Conversely, the data presented by Di Iorio et al. [26] were derived from self-administered questionnaires completed by 157 gynecologists who attended 15 scientific conferences. Nevertheless, data concerning the administration of the association of doxylamine succinate and 10 mg of pyridoxine hydrochloride reported in the two surveys are similar: 65.0% [26] vs. 66.4% in our survey. Therefore, the attitude towards prescription by gynecologists [26] was confirmed by the real-world data from pregnant women reporting NVP in our survey. This observation indicates that the fixed association of doxylamine succinate and 10 mg of pyridoxine hydrochloride, which was registered in Italy from 2019, is currently considered a valuable therapeutic option for treating NVP, in agreement with the guidelines established by the American College of Obstetricians and Gynecologists (ACOG) [26] and the findings of several studies [27,28].

The data also demonstrate that NVP is often undertreated, with only 24.6% of women with NVP receiving medication to alleviate their symptoms.

### 4.2. Strengths and Limitations

The limitations previously documented in the interim analysis [17] were mainly in relation to the limited sample size analyzed. First, the low number of women reporting severe symptoms in the interim analysis did not allow for the evaluation of the relationship between NVP symptoms and their severity, and between QOL and NVP severity; on the other hand, the sample size was adequate for these evaluations in this present final analysis. Second, the margin of error in the interim analysis was 7% (due to the sample size of 232 subjects); in the present final analysis, the margin of error was reduced to 4.2%.

The decision to conduct the study in only three large centers (in Northern, Central, and Southern Italy) was an objective limitation, potentially not being representative of all pregnant women in Italy. This decision was driven by the potential risks associated with involving centers that lacked experience and the potential for data fragmentation.

Another limitation is that, as the interviews were conducted after the 12-week, voluntary abortion window, the survey did not assess the number of women whose pregnancies were terminated due to NVP.

A strength was that the questionnaire was intentionally concise (nine postpartum items) to balance comprehensiveness with respondent burden. Items reflected current prescribing patterns and trends disseminated via social media. Wording was tailored for clarity and pretested to ensure comprehension. Closed-ended questions minimized ambiguity, even for sensitive topics such as family impact or subsequent pregnancies. Conditional pathways were limited to avoid confusion during routine clinical visits, thereby enhancing data reliability and respondent compliance.

Finally, the survey considered only medications and remedies legally available during the study period; thus, illicit self-administration could not be captured. This contrasts with findings by Vanderziel et al. [29], reporting cannabis use in 14% of 826 pregnant U.S. women, and Gómez-Ruiz et al. [30], detecting psychoactive substances (mainly cannabis, methamphetamine, cocaine) in 42.3% of 300 Mexican women. Such data underline the need for systematic substance-use monitoring in future NVP surveys.

## 5. Conclusions

The present findings indicate that the prevalence of NVP in Italy is consistent with data from previous investigations in other countries. There is significant evidence that NVP has the capacity to exert a negative influence on the quality of life experienced by pregnant women. This under-diagnosed maternal illness requires recognition, and it is suggested that pregnancy-related NVP screenings be made available. Furthermore, interventions that prioritize quality of life are strongly recommended.

## Figures and Tables

**Table 1:** PUQE (modified *).

1. On average in a day how often did you feel sick?	for more than 6 h (score = 5)	4–6 h (score = 4)	2–3 h (score = 3)	for a maximum of 1 h (score = 2)	for nothing (score = 1)
2. On average in a day how many times do you vomit or have vomited?	7 or more times (score = 5)	5–6 times (score = 4)	3–4 times (score = 3)	1–2 times (score = 2)	never (score = 1)
3. On average in a day how many times have you chocked without vomiting?	7 or more times (score = 5)	5–6 times (score = 4)	3–4 times (score = 3)	1–2 times (score = 2)	never (score = 1)

Score: ≤6: mild; 7–12: moderate; ≥13 severe. * [18].

**Table 2:** Demographic data by Italian Geographical Region for all subjects.

	Italian Geographical Region				
Characteristic	North *n* = 196 ^1^	Center *n* = 150 ^1^	South & Islands *n* = 186 ^1^	Total *n* = 532 ^1^	*p* ^2^
**Age, years**					0.164
Mean (SD)	33.2 (4.1)	32.5 (5.4)	32.2 (5.0)	32.7 (4.9)	
Median (IQR)	33 (31–36)	33 (29–36)	33 (29–35)	33 (30–36)	
Range	18–44	18–42	19–43	18–44	
**Age group, *n* (%)**					0.037 *
Under 25	4 (2.0)	9 (6.0)	15 (8.1)	28 (5.3)	
25–29	34 (17.3)	34 (22.7)	34 (18.3)	102 (19.2)	
30–39	145 (74.0)	90 (60.0)	124 (66.7)	359 (67.5)	
40 or over	13 (6.6)	17 (11.3)	13 (7.0)	43 (8.1)	
**Ethnic origin, *n* (%)**					0.007 **
Caucasian	196 (36.8)	150 (28.2)	186 (35.0)	532 (100.0)	
**City of residence population, *n* (%)**					<0.001 ***
<10,000	31 (15.8)	64 (42.7)	36 (19.4)	131 (24.6)	
10,000–100,000	50 (25.5)	72 (48.0)	77 (41.4)	199 (37.4)	
>100,000	115 (58.7)	14 (9.3)	73 (39.2)	202 (38.0)	
**Education, *n* (%)**					<0.001 ***
Primary or secondary school	54 (27.6)	87 (58.0)	119 (64.0)	260 (48.9)	
Professional qualification	9 (4.6)	6 (4.0)	10 (5.4)	25 (4.7)	
Bachelor’s degree	111 (56.6)	49 (32.7)	53 (28.5)	213 (40.0)	
Master’s Degree or PhD	22 (11.2)	8 (5.3)	4 (2.2)	34 (6.4)	
**Work status, *n* (%)**					<0.001 ***
Employed	182 (96.3)	92 (82.9)	122 (89.1)	396 (90.6)	
Unemployed	6 (3.2)	6 (5.4)	4 (2.9)	16 (3.7)	
Other (Student, etc.)	1 (0.5)	13 (11.7)	11 (8.0)	25 (5.7)	
Not declared	7	39	49	95	

^1^: Kruskal–Wallis test for numerical variables; Fisher’s exact test (2 × 2 tables)/Fisher–Freeman–Halton test (R × C tables) for categorical variables. ^2^: * *p* < 0.05; ** *p* < 0.01; *** *p* < 0.001.

**Table 3:** Pregnancy related data by Italian Geographical Region for all subjects.

	Italian Geographical Region				
Characteristic	North *n* = 196 ^1^	Center *n*= 150 ^1^	South & Islands *n* = 186 ^1^	Total *n* = 532 ^1^	*p* ^2^
**Is this your first pregnancy? N (%)**					0.029 *
Yes	116 (59.2)	76 (50.7)	85 (45.7)	277 (52.1)	
No	80 (40.8)	74 (49.3)	101 (54.3)	255 (47.9)	
**Parity, *n* (%)**					0.283
No	120 (61.2)	79 (52.7)	107 (57.5)	306 (57.5)	
Yes	76 (38.8)	71 (47.3)	79 (42.5)	226 (42.5)	
**Type of care provider, *n* (%)**					<0.001 ***
Private gynecologist	104 (53.1)	84 (56.0)	69 (37.1)	257 (48.3)	
Public gynecologist	79 (40.3)	64 (42.7)	113 (60.8)	256 (48.1)	
Midwife	13 (6.6)	2 (1.3)	4 (2.2)	19 (3.6)	
**Sex of the care provider, *n* (%)**					<0.001 ***
Female	177 (90.3)	90 (60.0)	126 (67.7)	393 (73.9)	
Male	19 (9.7)	60 (40.0)	60 (32.3)	139 (26.1)	
**Pregnancy week at the time of interview (from the last menstrual period or presumed date), *n* (%)**					0.397
Mean (SD)	20.3 (1.0)	20.3 (0.9)	20.4 (0.8)	20.3 (0.9)	
Median (IQR)	20 (20–21)	20 (20–21)	20 (20–21)	20 (20–21)	
Range	18–22	18–22	18–22	18–22	
**Number of children ^3^, Statistics**					0.102
Mean (SD)	0.4 (0.6)	0.7 (0.9)	0.6 (0.8)	0.6 (0.8)	
Median (IQR)	0 (0–1)	0 (0–1)	0 (0–1)	0 (0–1)	
Range	0–4	0–7	0–5	0–7	
**Number of children ^3^, *n* (%)**					0.070
0	120 (61.2)	79 (52.7)	106 (57.3)	305 (57.4)	
1	67 (34.2)	50 (33.3)	59 (31.9)	176 (33.1)	
2	7 (3.6)	18 (12.0)	16 (8.6)	41 (7.7)	
3	1 (0.5)	1 (0.7)	3 (1.6)	5 (0.9)	
4	1 (0.5)	1 (0.7)	0 (0.0)	2 (0.4)	
5	0 (0.0)	0 (0.0)	1 (0.5)	1 (0.2)	
7	0 (0.0)	1 (0.7)	0 (0.0)	1 (0.2)	

^1^: Kruskal–Wallis test for numerical variables; Fisher’s exact test (2 × 2 tables)/Fisher–Freeman–Halton test (R × C tables) for categorical variable. ^2^*:* * *p* < 0.05; *** *p* < 0.001; ^3^ one (1) non responder in South, islands region.

**Table 4:** NVP prevalence according to demographic data.

	Do You Suffer or Have You Suffered from Nausea and/or Vomiting During Current Pregnancy?					
Characteristic	Yes *n* = 353 ^1^	No *n* = 179 ^1^	Total *n* = 532 ^1^	*p* ^2^	NVP Prevalence Rate (Yes/ Total %)	*p* ^2^
**Age, years**				0.270		
Mean (SD)	32.8 (4.9)	32.5 (4.7)	32.7 (4.9)		66.4	
Median (IQR)	33 (30–36)	33 (29–35)	33 (30–36)			
Range	18–43	21–44	18–44			
**Age group, *n* (%)**				0.325		0.726
Under 25	20 (5.7)	8 (4.5)	28 (5.3)		71.4	
25–29	60 (17.0)	42 (23.5)	102 (19.2)		58.8	
30–39	245 (69.4)	114 (63.7)	359 (67.5)		68.2	
40 or over	28 (7.9)	15 (8.4)	43 (8.1)		65.1	
**Italian geographical region, *n* (%)**				0.635		0.915
North	129 (36.5)	67 (37.4)	196 (36.8)		65.8	
Center	96 (27.2)	54 (30.2)	150 (28.2)		64.0	
South & Islands	128 (36.3)	58 (32.4)	186 (35.0)		68.8	
**City of residence population, *n* (%)**				0.404		0.857
<10.000	85 (24.1)	46 (25.7)	131 (24.6)		64.9	
10.000–100.000	127 (36.0)	72 (40.2)	199 (37.4)		63.8	
>100.000	141 (39.9)	61 (34.1)	202 (38.0)		69.8	
**Education, *n* (%)**				0.545		0.508
Primary or secondary school	171 (48.4)	89 (49.7)	260 (48.9)		65.8	
Professional qualification	20 (5.7)	5 (2.8)	25 (4.7)		80.0	
Bachelor’s degree	140 (39.7)	73 (40.8)	213 (40.0)		65.7	
Master’s Degree or PhD	22 (6.2)	12 (6.7)	34 (6.4)		64.7	
**Work status, *n* (%)**				0.928		0.903
Employed	270 (90.9)	126 (90.0)	396 (90.6)		68.2	
Unemployed	11 (3.7)	5 (3.6)	16 (3.7)		68.8	
Other (Student, etc.)	16 (5.4)	9 (6.4)	25 (5.7)		64.0	
Not declared	56	39	95		-	

^1^: Kruskal-Wallis test for numerical variables; Fisher’s exact test (2 × 2 tables)/Fisher–Freeman–Halton test (R × C tables) for categorical variables ^2^.

**Table 5:** Duration of NVP symptoms and severity.

	PUQE Severity				
Characteristics	Mild *n* = 123 ^1^	Moderate *n* = 218 ^1^	Severe *n* = 12 ^1^	Total *n* = 353 ^1^	*p* ^2^
**When did the symptoms start?** **(indicate the week of onset of symptoms since the last menstruation)**					0.662
Mean (SD)	7.4 (3.2)	6.9 (2.6)	6.4 (1.7)	7.0 (2.8)	
Median (IQR)	6 (5–10)	6 (5–8)	6 (5–7)	6 (5–8)	
Range	4–20	4–18	4–10	4–20	
not declared	18	34	1	53	
**Are the symptoms still ongoing? *n* (%)**					0.016 *
Yes	26 (21.1)	76 (34.9)	5 (41.7)	107 (30.3)	
No	97 (78.9)	142 (65.1)	7 (58.3)	246 (69.7)	
**How long did the symptoms last (in weeks)? *n* (%)**					<0.001 ***
Mean (SD)	8.2 (4.6)	10.6 (5.3)	10.2 (4.1)	9.7 (5.1)	
Median (IQR)	7 (4–12)	10 (7–14)	11 (7–13)	9 (6–13)	
Range	1–21	2–46	4–16	1–46	
**How long did the symptoms last (in months)? *n* (%)**					0.001 **
Up to a month	31 (25.2)	29 (13.3)	2 (16.7)	62 (17.6)	
Up to 2 months	45 (36.6)	53 (24.3)	2 (16.7)	100 (28.3)	
Up to 3 months	24 (19.5)	63 (28.9)	3 (25.0)	90 (25.5)	
More than 3 months	23 (18.7)	73 (33.5)	5 (41.7)	101 (28.6)	
**Did the symptoms last beyond 12 weeks of pregnancy (first trimester)? *n* (%)**					<0.001 ***
Yes	54 (43.9)	159 (72.9)	9 (75.0)	222 (62.9)	
No	69 (56.1)	59 (27.1)	3 (25.0)	131 (37.1)	
**Did the symptoms last beyond the fourth month? *n* (%)**					0.399
Yes	32 (59.3)	88 (55.3)	7 (77.8)	127 (57.2)	
No	22 (40.7)	71 (44.7)	2 (22.2)	95 (42.8)	
Not declared	69	59	3	131	
**Did you have nausea/only vomiting/or both? *n* (%)**					<0.001 ***
Nausea only	87 (70.7)	62 (28.4)	0 (0.0)	149 (42.2)	
Vomiting only	6 (4.9)	5 (2.3)	0 (0.0)	11 (3.1)	
Nausea and vomiting	30 (24.4)	151 (69.3)	12 (100.0)	193 (54.7)	

^1^: Kruskal–Wallis test for numerical variables; Fisher’s exact test (2 × 2 tables)/Fisher–Freeman–Halton test (R × C tables) for categorical variables. ^2^: * *p* < 0.05; ** *p* < 0.01; *** *p* < 0.001.

**Table 6:** QOL by NVP severity (1st part).

	PUQE Severity				
Characteristic	Mild *n* = 123 ^1^	Moderate *n* = 218 ^1^	Severe *n* = 12 ^1^	Total *n* = 353 ^1^	*p* ^2^
**Did you ever have to go to the emergency room because of these symptoms? *n* (%)**					<0.001 ***
Yes	0 (0.0)	13 (6.0)	2 (16.7)	15 (4.2)	
No	123 (100.0)	205 (94.0)	10 (83.3)	338 (95.8)	
**Were you hospitalized because of these symptoms? *n* (%)**					0.057
Yes	0 (0.0)	2 (0.9)	1 (8.3)	3 (0.8)	
No	123 (100.0)	216 (99.1)	11 (91.7)	350 (99.2)	
**Were there any limitations on your daily activities due to these symptoms? *n* (%)**					<0.001 ***
None	68 (55.7)	66 (31.9)	1 (12.5)	135 (40.1)	
Partial limitations	48 (39.3)	97 (46.9)	1 (12.5)	146 (43.3)	
Many limitations	6 (4.9)	44 (21.3)	6 (75.0)	56 (16.6)	
Not declared	1	11	4	16	
**Have you ever thought about terminating your pregnancy because of these symptoms? *n* (%)**					0.568
Yes	0 (0.0)	2 (0.9)	0 (0.0)	2 (0.6)	
No	123 (100.0)	216 (99.1)	12 (100.0)	351 (99.4)	
**How did you feel about these symptoms? ^3^ (More than one answer allowed)** ***n* (%)**					0.114
I accepted because I knew it is normal in pregnancy	6 (21.5)	3 (10.7)	0 (0.0)	9 (32.2)	
Sad	2 (7.1)	5 (17.9)	0 (0.0)	7 (25.0)	
Depressed	2 (7.1)	3 (10.8)	0 (0.0)	5 (17.9)	
Agitated	0 (0.0)	1 (3.6)	0 (0.0)	1 (3.6)	
Worried	0 (0.0)	2 (7.1)	0 (0.0)	2 (7.1)	
Isolated	0 (0.0)	2 (7.1)	0 (0.0)	2 (7.1)	
Not understood by my family/partner	0 (0.0)	2 (7.1)	0 (0.0)	2 (7.1)	
Guilty	0 (0.0)	0 (0.0)	0 (0.0)	0 (0.0)	
No response				325	
**All**	10 (35.7)	18 (64.3)	0 (0.0)	28 (100)	

^1^: Kruskal–Wallis test for numerical variables; Fisher’s exact test (2 × 2 tables)/Fisher–Freeman–Halton test (R × C tables) for categorical variables. ^2^: *** *p* < 0.001. ^3^: Percents calculated from the total number of answers (28).

**Table 7:** QOL by NVP severity (2nd part).

	PUQE Severity				
Characteristic	Mild *n* = 123 ^1^	Moderate *n* = 218 ^1^	Severe *n*= 12 ^1^	Total *n* = 353 ^1^	*p* ^2^
**What was the impact of these symptoms on your relationship with your partner? *n* (%)**					<0.001 ***
None	81 (65.9)	114 (52.3)	3 (25.0)	198 (56.1)	
Slight impact	39 (31.7)	70 (32.1)	2 (16.7)	111 (31.4)	
Moderate impact	2 (1.6)	25 (11.5)	4 (33.3)	31 (8.8)	
Heavy impact	1 (0.8)	9 (4.1)	3 (25.0)	13 (3.7)	
**What was the impact of these symptoms on your social relationships? *n* (%)**					<0.001 ***
None	70 (56.9)	102 (46.8)	2 (16.7)	174 (49.3)	
Slight impact	43 (35.0)	70 (32.1)	3 (25.0)	116 (32.9)	
Moderate impact	8 (6.5)	34 (15.6)	2 (16.7)	44 (12.5)	
Heavy impact	2 (1.6)	12 (5.5)	5 (41.7)	19 (5.4)	
**What was the impact of these symptoms on your work activity? *n* (%)**					0.001 **
None	73 (59.3)	96 (44.0)	4 (33.3)	173 (49.0)	
Slight impact	34 (27.6)	59 (27.1)	2 (16.7)	95 (26.9)	
Moderate impact	10 (8.1)	39 (17.9)	1 (8.3)	50 (14.2)	
Heavy impact	6 (4.9)	24 (11.0)	5 (41.7)	35 (9.9)	
**Did you have to take time off from work because of these symptoms? *n* (%)**					0.017 *
No	103 (83.7)	158 (72.5)	6 (50.0)	267 (75.6)	
A few times	12 (9.8)	36 (16.5)	5 (41.7)	53 (15.0)	
Had to request early maternity leave	8 (6.5)	24 (11.0)	1 (8.3)	33 (9.3)	
**How much did these symptoms affect your eventual decision to have another child? *n* (%)**					0.020 *
Not at all	117 (95.1)	191 (87.6)	8 (66.7)	316 (89.5)	
A slight influence	4 (3.3)	12 (5.5)	2 (16.7)	18 (5.1)	
A moderate influence	2 (1.6)	9 (4.1)	1 (8.3)	12 (3.4)	
A strong influence	0 (0.0)	6 (2.8)	1 (8.3)	7 (2.0)	

^1^: Kruskal–Wallis test for numerical variables; Fisher’s exact test (2 × 2 tables)/Fisher–Freeman–Halton test (R × C tables) for categorical variables. ^2^: * *p* < 0.05; ** *p* < 0.01; *** *p* < 0.001

**Table 8:** Characteristics of newborn and perinatal outcomes for NVP severity in the 466 women which completed the postpartum questionnaire. The table shows the distribution of pregnancy-related outcomes across PUQE severity categories within the NVP population and in women without NVP.

	Do You Suffer or Have You Suffered from Nausea and/or Vomiting During Current Pregnancy?						
	Yes					No	
Characteristic	PUQE Mild	PUQE Moderate	PUQE Severe	Total *n* = 313 ^1^	*p* ^3^	Total *n* = 153	*p* ^2^
	*n* = 123 ^1^	*n* = 218 ^1^	*n* = 12 ^1^				
**Sex of the newborn, *n* (%)**					0.193		
Female	52 (48.1)	103 (53.4)	9 (75.0)	164 (52.4)		80 (52.3)	
Male	56 (51.9)	90 (46.6)	3 (25.0)	149 (47.6)		73 (47.7)	
**Birth term, N (%)**					0.499		
Pre term	8 (7.5)	22 (11.5)	0 (0.0)	30 (9.7)		11 (7.4)	
Full term	98 (91.6)	168 (88.0)	12 (100.0)	278 (89.7)		137 (91.9)	
Post term	1 (0.9)	1 (0.5)	0 (0.0)	2 (0.6)		1 (0.7)	
Not declared	1	2	0	3		4	
**Gestational age (in days)**					0.142		
Mean (SD)	274.3 (13.9)	273.3 (12.4)	279.3 (7.1)	273.9 (12.8)		274.0 (12.3)	
Median (IQR)	277 (269–282)	276 (267–282)	281 (275–285)	276 (268–282)		276 (269–282)	
Range	191–294	215–294	264–290	191–294		191–294	
Not declared	1	2	0	3		4	
**Weight at birth (kg)**					0.046 *		
Mean (SD)	3.2 (0.6)	3.2 (0.5)	3.5 (0.4)	3.2 (0.5)		3.2 (0.5)	
Median (IQR)	3 (3–3)	3 (3–4)	4 (3–4)	3 (3–4)		3 (3–4)	
Range	1–5	1–5	3–4	1–5		1–5	
**Complications, *n* (%)**					0.098		
No	85 (78.7)	129 (66.8)	9 (75.0)	223 (71.2)		104 (68.0)	
Yes	23 (21.3)	64 (33.2)	3 (25.0)	90 (28.8)		49 (32.0)	
**List of mentioned complications and %**					0.001 *** ^2^		
Gestational diabetes (%)	10 (31.2)	20 (62.5)	2 (6.3)	32 (35.6) (100.0)		23 (46.8)	0.52 ^ns^
Fetal growth restriction (%)	3 (27.2)	8 (72.8)	0 (0.0)	11 (12.2) (100.0)		9 (18.4)	
Gestational hypothyroidism (%)	0 (0.0)	6 (85.7)	1 (14.3)	7 (7.8) (100.0)		2 (4.1)	
Hypertension (%)	4 (36.4)	6 (54.5)	1 (9.1)	11 (12.2) (100.0)		7 (14.3)	
Pre-eclampsia (%)	0 (0.0)	4 (80.0)	1 (20.00)	5 (5.6) (100.0)		2 (4.1)	
Post-partum hemorrhage (%)	0 (0.0)	4 (100.0)	0 (0.0)	4 (4.4) (100.0)		2 (4.1)	
Other (specified) (%)	5 (26.3)	13 (68.4)	1 (5.3)	19 (21.15) (100.0)		4 (8.2)	
Other (not indicated) (%)	1 (100.0)	0 (0.0)	0 (0.0)	1 (1.1) (100.0)		0 (0.0)	
**Total**	23 (25.5)	61 (67.8)	6 (6.7)	90 (100.0) (100.0)		49 (100.0)	

^1^: Kruskal–Wallis test for numerical variables; Fisher’s exact test (2 × 2 tables)/Fisher–Freeman–Halton test (R × C tables) for categorical variables. ^2^: Chi-square test. ^3^: * *p* < 0.05; *** *p* < 0.001. ^ns^: *p* = Not significant.

## Data Availability

The Statistical Analysis Plan and the study protocol can be sent upon request to the authors.

## Supplementary Materials

The following supporting information can be downloaded at https://www.mdpi.com/article/10.3390/jcm15041365/s1: Table S1: Questionnaire for pregnancy period (English Version); Table S2: Questionnaire for post-pregnancy (English Version).

## Abbreviations

The following abbreviations are used in this manuscript:

ACOG	American College of Obstetricians and Gynecologists
EC	Ethics Committees
EDC	Electronic Data Collection
GCP	Good Clinical Practice
GDPR	General Data Protection Regulation
HIPAA	Health Insurance Portability and Accountability Act
ICH	International Council for Harmonisation
IQR	Interquartile Range
ITT	Intention to Treat
NVP	Nausea and vomiting of pregnancy
NCT	National Clinical Trial
PAPI	Paper-and-pen interview
PP	Per Protocol
PUQE	Pregnancy-Unique Quantification of Emesis
QOL	Quality of life
SD	Standard Deviation
SOC	Service Organization Control
SSAE	Statements on Standards for Attestation Engagements
