# Supplementary material for: Prevalence and Burden of Nausea and Vomiting in Pregnant Women: Final Analysis of the PURITY Survey
Source: J Clin Med. 2026 Feb 9;15(4):1365. doi: 10.3390/jcm15041365 (PMC12942206; doi:10.3390/jcm15041365)
Supplement: Supplementary file 1 [file jcm-15-01365-s001.zip › Table S1 Questionnaire for pregnancy period (English Verison).pdf]

QUESTIONNAIRE FOR PREGNANT WOMEN

Prevalence and Burden of Nausea and vomiting in pregnant women. An Italian Survey (PURITY)

Demographic data

* Required field

1. Date of interview (dd/mm/yy) *

2. Sequential number of the woman interviewed *

3. Year of birth (yyyy) *

4. Geographical area of residence *

   - ○ north
   - ○ center
   - ○ South, islands

5. City of residence *

   - ○ > 100,000 inhabitants
   - ○ between 99,000 and 10,000 inhabitants
   - ○ < 10,000 inhabitants

6. a) Country of origin *

   b) Ethnicity*
   - ○ Caucasian
   - ○ non-Caucasian - **go to point 44**

7. Educational qualification *

- ○ lower secondary school diploma
- ○ vocational qualification
- ○ high school diploma
- ○ Bachelor's degree
- ○ Master's degree or degree under the old system
- ○ university master's degree
- ○ doctorate
- ○ other ______

8. Profession (see attached tables with ISTAT categories) *

______

9. Is this your first pregnancy? *

- ○ YES - **go to step 11**
- ○ NO

10. **Only if you answered NO to question 9:** How many children have you had previously?

______

11. Type of current pregnancy: *

- ○ spontaneous twin pregnancy - **go to point 44**
- ○ spontaneous single
- ○ medically assisted reproduction (MAR) twin - **go to question 44**
- ○ medically assisted reproduction (MAR) single - **go to question 44**

12. Week of pregnancy at the time of the interview calculated from the last menstrual period or the expected date of delivery*
(if less than 18 weeks or more than 22 weeks, go to point 44)

______

- ○ calculated from last menstrual period
- ○ calculated from the expected date of delivery

13. Type of healthcare provider: *

- ○ private gynecologist
- ○ public gynecologist
- ○ midwife

14. Gender of healthcare provider *

- ○ male
- ○ female

FIRST INTERVIEW (conducted face to face in hospital)

15. Do you suffer or have you suffered from nausea and/or vomiting during pregnancy? *

- ○ YES
- ○ NO - **go to question 32**

16. **Only if you answered YES to question 15:** When did the symptoms start? (indicate the week of onset of symptoms from your last menstrual period) *

[ ]

17. **Only if you answered YES to question 15:** Are the symptoms still present? *

- ○ YES
- ○ NO

18. **Only if you answered YES to points r 15**: How long did the symptoms last (in weeks)? *

[ ]

19. **Only if you answered YES to question 15**: Did the symptoms last beyond 12 weeks of pregnancy (first trimester)? *

- ○ YES
- ○ NO

20. **Only if you answered YES to question 19**: Did the symptoms last beyond the fourth month? *

- ○ YES
- ○ NO

21. **Only if you answered YES to question 15**: Did you experience nausea only/vomiting only/or both? *

- ○ nausea
- ○ vomiting
- ○ vomiting and nausea

Assessment of severity (PUQE score). We will ask you some questions about nausea and/or vomiting symptoms during pregnancy

22. Have you ever had to go to the emergency room because of these symptoms? *

- ○ YES
- ○ NO

23. Have you ever been hospitalized because of these symptoms? *

- ○ YES
- ○ NO

24. Have these symptoms limited your ability to carry out your daily activities? *

○	○	○	○
No	partially limited	very limited	prevent you

25. What impact did these symptoms have on your relationship with your partner? *

○	○	○	○
None	Slightly affected	Moderately affected	Significantly affected

26. What impact have these symptoms had on your social relationships? *

○	○	○	○
None	Slightly affected	Moderately affected	Significantly affected

27. What impact have these symptoms had on your work? *

○	○	○	○
None	Slightly affected	Moderately affected	Significantly affected

28. Did you have to take time off work because of these symptoms? *

- ○ No
- ○ sometimes
- ○ I had to request early maternity leave

29. How much did these symptoms influence your decision to have another child? *

- ○ Not at all
- ○ They had a slight impact
- ○ They had a moderate impact
- ○ They had a significant impact

30. Did you ever consider terminating the pregnancy because of these symptoms? *

- ○ YES
- ○ NO

31. How did these symptoms make you feel? (you can select more than one answer)*

- ○ Sad
- ○ depressed
- ○ agitated
- ○ worried
- ○ isolated
- ○ not understood by my family and/or partner
- ○ feeling guilty towards my family and/or workplace
- ○ I accepted it because I knew it was normal during pregnancy

32. Have you ever discussed the problem of nausea and vomiting and how to manage it with your gynecologist or midwife? *

- ○ NO - **go to question 34**
- ○ YES - I asked for information
- ○ YES - they brought it up first: **(specify)**
  - ○ My gynecologist
  - ○ My gynecologist
  - ○ My midwife
  - ○ My obstetrician

33. What were you told? (you can select more than one answer) *

- ○ I was told to be patient and that it will pass

- ○ I was given dietary advice
- ○ I was advised to take multivitamins
- ○ I was advised to use ginger
- ○ I was advised to use acupuncture
- ○ I was advised to use Sea Band (elastic acupressure bracelet)

34. **Only if you answered NO to question 32:** If you did not discuss this topic with your gynecologist/obstetrician, how did you behave? *

- ○ I put up with it because I know it's normal
- ○ I asked for advice from [ ]
- ○ I searched for information on the internet
- ○ I asked in a chat room to see how to behave, what to use

35. Did you use natural remedies for nausea and/or vomiting? *

- ○ YES - specify how many: [ ]
- ○ NO - **go to question 37**

36. **Only if you answered YES to question 35:** What is your opinion of the natural substances/remedies you took? *

- ○ They did not help at all
- ○ I had a slight improvement
- ○ I had a moderate improvement
- ○ The symptoms went away

37. Did your doctor or gynecologist prescribe any medication (excluding natural remedies) for nausea and/or vomiting? *

- ○ YES
- ○ NO - **specify reason and go to question 41**: [ ]

38. **Only if you answered YES to question 37:** Did you take these medications for nausea and/or vomiting at the dose prescribed by your gynecologist? *

- ○ YES

○ NO **- specify reason and go to question 41:**

- ○ I was afraid for my baby
- ○ I took a lower dose
- ○ Other

39. **Only if you answered YES to question 38:** In your opinion, was it helpful to take these medications? *

- ○ They did not help at all
- ○ I felt/feel better, but I feel/felt guilty/worried about my baby
- ○ I had a slight improvement
- ○ I have had a moderate improvement
- ○ the symptoms have gone away

40. **Only if you answered YES to question 38:** Indicate (if possible) the name of the medication or medications if there is more than one

- ○ Biochetasi®
- ○ Plasil®
- ○ Torecan®
- ○ Nuperal®
- ○ Stemetil®
- ○ Other

Assessment of symptom severity during pregnancy with nausea and/or vomiting

41. On average, how long do you experience or have you experienced nausea and/or stomach discomfort in a day? *

- ○ Not at all
- ○ For a maximum of 1 hour
- ○ 2-3 hours
- ○ 4-6 hours
- ○ For more than 6 hours

42. On average, how many times did you vomit in a day? *

- ○ Never
- ○ 1-2 times
- ○ 3-4 times
- ○ 5-6 times
- ○ 7 or more times

43. On average, how many times a day did you feel like vomiting without actually vomiting? *

- ○ Never
- ○ 1-2 times
- ○ 3-4 times
- ○ 5-6 times
- ○ 7 or more times

44. Other comments (specify whether from the compiler or the patient interviewed)

45. Compiler's statement

First and last name

○ I confirm that the information provided is accurate and corresponds to the responses of the patients interviewed.
