# Supplementary material for: Prevalence and Burden of Nausea and Vomiting in Pregnant Women: Final Analysis of the PURITY Survey
Source: J Clin Med. 2026 Feb 9;15(4):1365. doi: 10.3390/jcm15041365 (PMC12942206; doi:10.3390/jcm15041365)
Supplement: Supplementary file 1 [file jcm-15-01365-s001.zip › Table S2 Questionnaire for post-pregnancy (English Verison).pdf]

QUESTIONNAIRE FOR WOMEN AFTER PREGNANCY

Prevalence and Burden of Nausea and vomiting in pregnant women. An Italian Survey (PURITY)

SECOND INTERVIEW (FOLLOW-UP)

* Mandatory field

1. Date of interview (dd/mm/yy) *

2. Subject number *

3. Date of birth *

4. Sex of newborn *

   ○ male
   ○ female

5. Newborn weight [kg] *

6. Gestational age at birth - specify number of weeks + number of days (e.g., 37 weeks + 3 days) *

   - weeks
   +
   - days

7. Any complications: *

- ○ NO
- ○ YES

8. If YES, specify:

- ○ gestational diabetes
- ○ hypertension
- ○ pre-eclampsia
- ○ fetal growth restriction
- ○ other ____

9. Other comments (specify whether from the compiler or the patient interviewed)

____

10. Compiler's statement.

First and last name ____

○ I confirm that the information provided is accurate and corresponds to the responses of the patients interviewed.
